# Meta-analysis of the space flight and microgravity response of the Arabidopsis plant transcriptome

**DOI:** 10.1038/s41526-023-00247-6

**Published:** 2023-03-20

**Authors:** Richard Barker, Colin P. S. Kruse, Christina Johnson, Amanda Saravia-Butler, Homer Fogle, Hyun-Seok Chang, Ralph Møller Trane, Noah Kinscherf, Alicia Villacampa, Aránzazu Manzano, Raúl Herranz, Laurence B. Davin, Norman G. Lewis, Imara Perera, Chris Wolverton, Parul Gupta, Pankaj Jaiswal, Sigrid S. Reinsch, Sarah Wyatt, Simon Gilroy

**Affiliations:** 1 Department of Botany, University of Wisconsin, Madison, WI 53706 USA; 2 Los Alamos National Laboratory, Bioscience Division, Los Alamos, NM 87545 USA; 3 NASA John F. Kennedy Space Center, Titusville, FL 32899 USA; 4 Space Biosciences Division, NASA Ames Research Center, Moffett Field, CA 94035 USA; 5 Logyx, LLC, Mountain View, CA 94043 USA; 6 Bionetics, Yorktown, VA 23693 USA; 7 Department of Statistics, University of Wisconsin, Madison, WI 53706 USA; 8 Centro de Investigaciones Biológicas Margarita Salas (CSIC), 28040 Madrid, Spain; 9 Institute of Biological Chemistry, Washington State University, Pullman, WA 99164-741 USA; 10 Department of Plant and Microbial Biology, North Carolina State University, Raleigh, NC 27695 USA; 11 Department of Botany and Microbiology, Ohio Wesleyan University, Delaware, OH 43015 USA; 12 Department of Botany and Plant Pathology, Oregon State University, Corvallis, OR 97331 USA; 13 Department of Environmental and Plant Biology, Ohio University, Athens, OH 45701 USA

**Keywords:** Plant sciences, Genetic databases

## Abstract

Spaceflight presents a multifaceted environment for plants, combining the effects on growth of many stressors and factors including altered gravity, the influence of experiment hardware, and increased radiation exposure. To help understand the plant response to this complex suite of factors this study compared transcriptomic analysis of 15 *Arabidopsis thaliana* spaceflight experiments deposited in the National Aeronautics and Space Administration’s GeneLab data repository. These data were reanalyzed for genes showing significant differential expression in spaceflight versus ground controls using a single common computational pipeline for either the microarray or the RNA-seq datasets. Such a standardized approach to analysis should greatly increase the robustness of comparisons made between datasets. This analysis was coupled with extensive cross-referencing to a curated matrix of metadata associated with these experiments. Our study reveals that factors such as analysis type (i.e., microarray versus RNA-seq) or environmental and hardware conditions have important confounding effects on comparisons seeking to define plant reactions to spaceflight. The metadata matrix allows selection of studies with high similarity scores, i.e., that share multiple elements of experimental design, such as plant age or flight hardware. Comparisons between these studies then helps reduce the complexity in drawing conclusions arising from comparisons made between experiments with very different designs.

## Introduction

Spaceflight imposes a unique suite of environmental effects on biology. For example, microgravity severely curtails the signals normally generated on Earth from the intrinsic weight of a plant’s organs^1^ and by its gravity perceptive cells^2–4^. By contrast, in the terrestrial environment, these are key factors driving normal growth and development. In addition, gravitational forces on Earth govern a host of physical processes including gas and liquid flow that are important for normal plant function. Thus, the microgravity environment can lead to the development of anoxic regions around metabolically active plant tissues and altered patterns of evaporative and convective cooling that can impact leaf function and physiology^5–8^. Additionally, the increased radiation exposure inherent in spaceflight is likely to trigger its own array of responses within the plant. The combination of these spaceflight-linked effects is outside the evolutionary history of terrestrial biology and so it remains complicated to predict the effects of spaceflight on organisms. Yet, understanding the molecular and physiological responses of plants to these conditions remains an important goal for space biologists, not the least because plants are integral to many plans for life support on long-duration crewed missions and for colonization^9^.

One way to probe the responses of organisms to spaceflight is by analysis of changes in their transcriptomes, proteomes, metabolomes, genomes and epigenomes induced by exposure to this environment. In the field of plant biology the National Aeronautics and Space Administration’s (NASA’s) GeneLab data repository^10,11^ has aggregated many such omics datasets. Critically, the deposited data are associated with extensive metadata covering elements of each experiment’s design ranging from features of the hardware, radiation exposure and lighting regime to treatment duration, genotype and organism age. Such extensive and accurate metadata are critical to understanding the breadth of differences in experimental designs when making comparisons between studies. This insight is important as the flight hardware used, the analysis methodology employed (e.g., microarray versus RNA-seq for transcriptome studies) and other experimental parameters likely superimpose their own, often poorly defined, influences on the results (so-called batch effects^12^). Indeed, recent analysis of rodent spaceflight data suggests differences in sample preservation eclipsed spaceflight-driven differences in mouse transcriptional profiling^13^. However, given the relatively few opportunities to conduct experiments in space, making comparisons between existing studies represents a potentially powerful approach to identify common responses in the often-limited available spaceflight data.

We have therefore imported 15 spaceflight-related plant transcriptome datasets from the GeneLab data repository and manually curated the associated metadata to develop a metadata matrix (hereafter, the Matrix). This approach allows the more robust design of comparisons between studies that share commonalities in experimental design. Our meta-analyses broadly confirmed the spaceflight-related changes in cell wall processes and oxidative stress that were highlighted in many of the original publications associated with each individual study. Additionally, Matrix-driven analyses helped reveal new response elements, such as conserved spaceflight-triggered changes in expression of the cold response gene *COLD RESPONSIVE 78* (*COR78*), and likely shifts in ion transport processes. We also identified factors within the experimental design such as choice of flight hardware and especially assay technique (i.e., microarray versus RNA-seq) that can impose greater differences between datasets than the spaceflight treatment. Thus, the Matrix allows researchers to explore the wealth of plant biology transcriptomic data generated during spaceflight-related studies and provides an approach to better understand underlying factors impacting the robustness of comparisons made between the different datasets.

## Results and discussion

### Comparative transcriptomics of plant spaceflight-response data

**Fig. 1:**
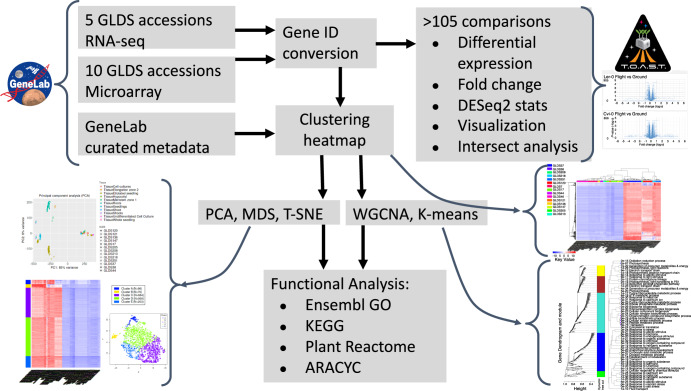
Uniform analysis pipeline applied to Arabidopsis GLDS datasets used in this study. Normalized expression arrays are imported from NASA’s GeneLab repository (https://genelab-data.ndc.nasa.gov/genelab/projects) and then parsed by the TOAST X-Species Transcriptional Explorer (https://astrobiology.botany.wisc.edu/x-species-astrobiology-genelab) for analysis of common features between experiments (cross experiment intersect analysis). The iDEP.92 R-shiny app^59^ is then used to generate expression heatmaps for clustering, and to perform Principal Component Analysis (PCA), Multidimensional Scaling analysis (MDS), t-distributed Stochastic Neighbor Embedding (T-SNE), Weighted Gene Correlation Network Analysis (WGCNA) and K-means statistical analyses. Functional analyses are then performed using the online tools at Ensembl GO^53^, KEGG (Kyoto Encyclopedia of Gene and Genomes)^56^, AraCyc^57^ and Reactome^58^. These data are then visualized as tables and dendrograms of the enriched functional groups that are altered by spaceflight and/or related stimuli.

One method to assess the similarities and differences in transcriptome-level responses between different plant spaceflight experiments is to make comparisons using the results of the analyses already presented in the primary literature on each study. This approach can be further expedited using tools, such as the Test of Arabidopsis Space Transcriptome (TOAST) database^14^ that aggregates these analyses into an interactive data exploration environment. Such comparative studies capitalize upon the unique insights of the researchers who performed each experiment and the tailored analytical tools and approaches they then employed to define differentially expressed genes (DEGs) in their original publications. We will refer to these studies as in-house analyses. However, the wide range of analytical pipelines used in such a primary literature-based approach inevitably imposes some limitations on the robustness of any conclusions that can be drawn between studies. This problem arises because differences in gene expression patterns between datasets likely involve both the effects of experimental treatments, such as growing plants in spaceflight versus a ground control, and of elements specific to the different analytical programs and statistical approaches used to analyze the data. Indeed, differences between results from different software packages analyzing the same raw transcriptomics datasets are well-documented in the literature^15^. Therefore, a complementary methodology was also applied by reanalyzing the plant studies used in our analysis via the common computational pipelines summarized in Fig. 1. A similar strategy of reanalyzing published datasets using a common computational approach has been used in the EMBL-EBI gene Expression Atlas. For example, when these researchers import RNA-seq data, a standardized analysis is performed using the integrated RNA-seq Analysis Pipeline, or iRAP, approach^16^. Although this analysis pipeline is different from the one we have adopted, the standardizing of analysis across all datasets for the EMBL-EBI gene Expression Atlas is designed with the same goal in mind: to reduce the potential for generating artifacts that are caused by making comparisons between datasets that have been the subject of different initial data analysis methodologies.

**Fig. 2:**
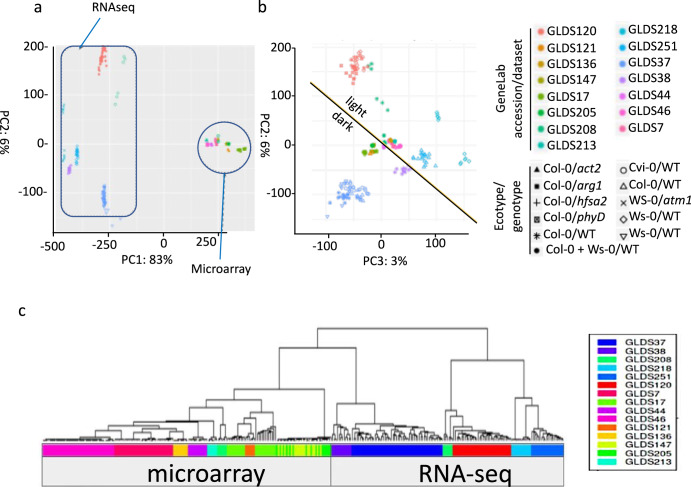
Principal component analysis (PCA) of the 15 plant datasets in the Matrix reveals clustering based on analystical approach (microarray versus RNA-seq) and by lighting conditions. Principal components sperate datasets by **a** microarray versus RNA-seq-based analyses and **b** by growth in the light versus the dark environment of the growth hardware. PC1 principal component 1, PC2 principal component 2, PC3 principal component 3. Percentage reflects the degree of variance accounted for by each principal component. **c** Euclidian hierarchical clustering confirms grouping by assay type (microarray versus RNA-seq) as major factor within the data. Ecotypes: Col Columbia, Cvi Cape Verde Island, Ws Wassilewskija, Col-0 + Ws, mixed sample 80% Ws, and 20% Col ecotypes. Genotypes: WT wild-type, *act2 actin 2*, *arg1 altered response to gravity 1, atm1* *ataxia-telangiectasia mutated 1*, *hsfa2 heat shock transcription factor A2, phyD* *phytochrome D*.

Using our common analysis pipeline approach to comparing DEGs across all the Arabidopsis studies, batch effects (i.e., confounding variables imposing effects on patterns of gene expression over and above those of the spaceflight treatment) became readily evident. Thus, Principal Component Analysis (PCA) and Euclidean hierarchical clustering revealed that rather than the comparison between of spaceflight and ground control, whether RNA-seq or microarray was used to detect patterns of gene expression is the factor with the largest effect on separating studies (PCA1, explaining 83% of the variance between experiments; Fig. 2a, c). Similar analysis showed the important but lesser impact of lighting environment (Fig. 2b). It is important to note here that we have used a statistical threshold of *p* < 0.01 to define a DEG. Our analysis pipeline also generates the more stringent adjusted *p*-value (or *q*-value) that corrects the *p*-value for the false discovery rate associated with multiple testing. Although we have analyzed the *p*-value filtered results to encompass as broad a set of DEGs as possible, *q*-values are presented in the tables of Supplementary Data, to allow the reader to define DEG lists using this parameter. Similarly, a cut off related to fold-change in expression (such as only evaluating genes showing ≥2-fold change in e.g., spaceflight versus their paired ground control) is often used in the literature to limit the extent of the gene lists being analyzed. Again, we have opted not to apply such a fold-change cut off to maintain the most inclusive list of DEGs for analysis. However, fold-change in expression level is also presented in the Supplementary Data, allowing the reader to filter the gene lists using a fold-change cut off as appropriate for their analyses

**Fig. 3:**
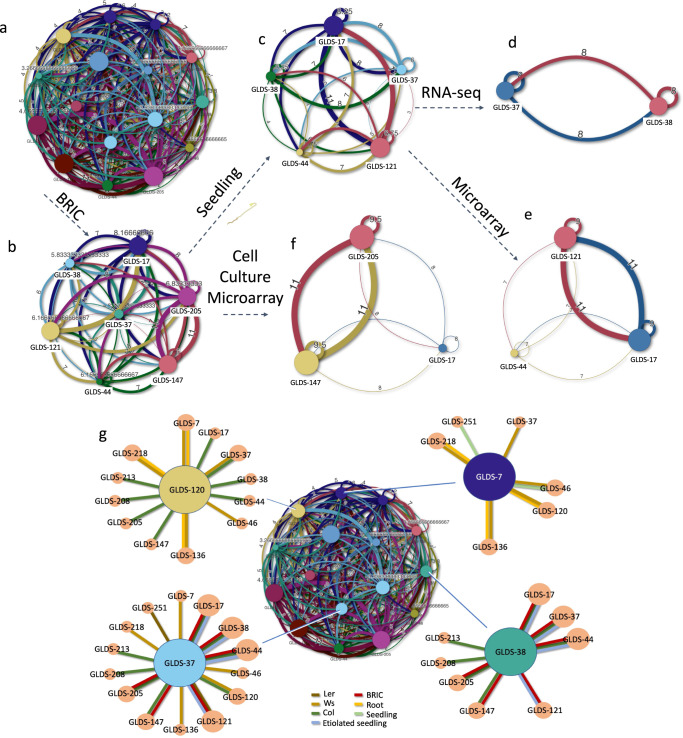
Pairwise factor correlation analysis creates a weighted network linking studies based on metadata similarity score. **a** Whole connectivity network. Numbers and thickness of connection (network edge) reflect degree of connectivity through shared metadata factors. **b**–**f** 5 sub-networks based on common BRIC hardware experiment design: **b** sub-network of experiments performed using the BRIC hardware (mean connectivity score: 6.3), **c** BRIC experiments involving seedlings (mean connectivity score: 6.0). Seedling experiments analyzed using **d** RNA-seq (mean connectivity score: 8) or **e** microarray (mean connectivity score: 7.6) and **f** BRIC experiments that have used cell cultures, all analyzed by microarray (mean connectivity score: 7.4). For **a**–**g** size of circle for each study reflects the number of connected factors available for pairwise comparison. **g** Examples of connectivity of GLDS-7, GLDS-37, GLDS-38, and GLDS-120 by tissues sampled and ecotypes analyzed. Colored lines reflect factor connecting studies. Ecotypes: Col, Columbia; Ws, Wassilewskija; Ler, Landsberg. See Supplementary Data 2 for full connectivity matrix.

**Fig. 4:**
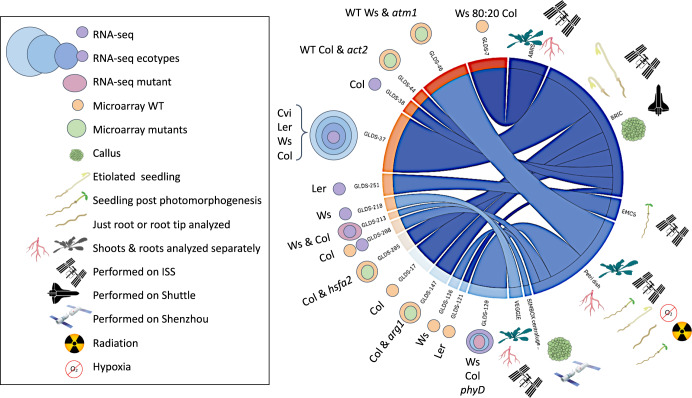
Graphical representation of metadata related to tissues, assay type and flight vehicle. The specific assay and tissue types for each dataset are indicated with network clustering based on hardware. See Supplementary Data 1 and 2 for the Matrix driving this visualization. Note the hardware used to analyze plant response to spaceflight often defines the types of tissue that are available and so these two variables are often linked. Purple color circles represent RNAseq analysis of wild-type Col-0 plants, shades of blue represent other WT ecotypes, the pink circle represents RNA-seq analysis performed on mutants. The size of circles is a qualitative representation of the amount of differentially expressed loci relative to other genetic varieties used during that study. Ecotypes: Col Columbia, Cvi Cape Verde Island, Ws Wassilewskija, Ler Landsberg, Col-0 + Ws mixed sample 80% Ws and 20% Col ecotypes. Genotypes: WT wild-type, *arg1 altered response to gravity 1, hsfa2 heat shock transcription factor A2, atm1* *ataxia-telangiectasia mutated 1*, *phyD* *phytochrome D*, Hardware: BRIC Biological Research in Canister, EMCS European Modular Cultivation System, VEGGIE Vegetable production system, SIMBOX SIMBOX incubator system, ABRS Advanced Biological Research System. An interactive version of this visualization is available at: https://gilroy-qlik.botany.wisc.edu/a/sense/app/20aa802b-6915-4b1a-87bd-c029a1812e2b/sheet/6241e71a-a3c5-4c63-9210-e05c743699d7/state/analysis.

We next created a connectivity network visualization system using all the pairwise comparisons that can be made between the GLDS used in our analysis (Fig. 3a–g; Supplementary Data 2; an interactive version of this connectivity analysis is available at: https://gilroy-qlik.botany.wisc.edu/a/sense/app/20aa802b-6915-4b1a-87bd-c029a1812e2b/sheet/6241e71a-a3c5-4c63-9210-e05c743699d7/state/analysis). Pairwise factor correlation analysis was performed by inspection of the Matrix in Supplementary Data 1 and manually scoring factors that are similar between different pairs of studies, assigning a value of 1 for each factor shared between a pair and a value of zero if that factor was different. Thus, the more factors in common between a pair of studies, the greater their similarity scores. The full pairwise similarity matrix can be found in Supplementary Data 2. This approach linked the studies using similarity scores reflecting commonalities in the different experimental designs and metadata factors within the datasets in our study. Such network analysis allowed us to further visualize and dissect links between the factors that potentially cause the clustering of studies identified in the PCA (Fig. 2). When represented graphically as links between studies and metadata factors, this analysis demonstrated that hardware and its associated lighting regimes were indeed likely key components that influence clustering of responses in the data (Fig. 4).

It is important to remember here that the lighting environment for an experiment is often dictated by the hardware that was used, for example, most plant experiments performed to date using the Biological Research in Canister (BRIC) hardware are conducted in the dark. Therefore, lighting and hardware are inevitably closely linked in our network analyses of current datasets. This observation also highlights the insight that could be gained by performing more studies that use the same hardware but with a range of lighting environments. Such analyses could help separate hardware-related effects on the plant during growth in space from those specifically triggered by the lighting environment under those conditions.

Such network analyses distinguish those studies sharing a high degree of network linkages within the Matrix, i.e., studies with a larger number of common features in their experimental design. Results of comparisons between such highly connected studies are candidates for more robust analyses due to these shared factors. For example, although the overall experimental designs behind GLDS-7, GLDS-37, GLDS-38, and GLDS-120 differ from each other by ecotype, hardware or experiment duration, each links to multiple other spaceflight experiments within the Matrix and form hubs in networks related to hardware and/or tissue sample type (Fig. 3g). Thus, GLDS-7 was performed in the Advanced Biological Research System (ABRS) and is of interest due to the high number of tissues and ecotypes in its experimental design that link to many other studies in the Matrix. GLDS-37 was conducted in the BRIC hardware and is extensively linked to other studies due to the large number of Arabidopsis ecotypes analyzed, as well as the many other Arabidopsis BRIC experiments available for comparison. GLDS-38 (BRIC) provides RNA-seq and paired proteomics data that likewise connect to many other BRIC datasets. GLDS-120 took place with a unique hardware setup (square Petri plates that were attached to the inside wall of the International Space Station), but contains multiple ecotypes, genotypes and light treatments that link it to many other studies in the Matrix. These connections to other studies suggest that comparisons within the local networks where each study acts as a hub are likely to be fruitful targets to extract common spaceflight-related responses.

Conversely, such network analyses also revealed studies that are the most distinct (i.e., least shared metadata factors with other Matrix studies). One clear set of such studies are those designed around terrestrial spaceflight analogs such as GLDS-46, GLDS-136, and GLDS-144. These experiments use elements such as hyperbaric chambers, space radiation analog exposures, and microgravity simulation on clinostats and random positioning machines to mimic specific aspects of the spaceflight environment and so are more distant in design to the other spaceflight experiments. Thus, as shown in Supplementary Fig. 1, pairwise similarity matrix comparisons show spaceflight studies are most similar to other spaceflight studies (average pairwise similarity score of 5.88 ± 1.93) and significantly less similar (*p* < 0.01) when compared to ground analog studies (average similarity 4.21 ± 1.36), which are most similar to other ground analog studies. This Matrix-driven network visualization then highlights the opportunity to design follow-up experiments that use these analogs of putative spaceflight stressors but where the design of the study is more interconnected to the factors seen in their closest spaceflight studies within the Matrix. Such aligned experimental designs could help increase the robustness of subsequent comparisons to the existing spaceflight data.

We next asked if we could define factors within the metadata other than spaceflight treatment that help define clustering within the studies. We therefore took the expression level data for each individual sample replicate (normalized probe fluorescence intensity for microarray and FPKM for RNAseq) from all the studies in the Matrix and calculated the Pearson’s correlation coefficient for all possible pairwise combinations (Supplementary Data 3). We next calculated the average Pearson’s correlation coefficient from this analysis for each set of replicates within an experiment, providing a measure of correlation for each treatment within a dataset to all other treatments in all datasets in the Matrix. We then sorted these data by each metadata factor within the Matrix to ask if a particular metadata factor stood out as explaining the patterns of correlation within the transcriptomics data. Of these, radiation treatment was the most highly correlated factor (Supplementary Data 3), followed by genotype, tissue/developmental stage, flight hardware and then altered gravity (i.e., spaceflight). This analysis again highlights the likelihood that many experimental factors are imposing patterns on spaceflight transcriptional profiles and reinforces the effects of radiation exposure as a key area for future spaceflight-related experimentation.

### Mining the Matrix for common patterns of spaceflight-responsive gene expression

**Fig. 5:**
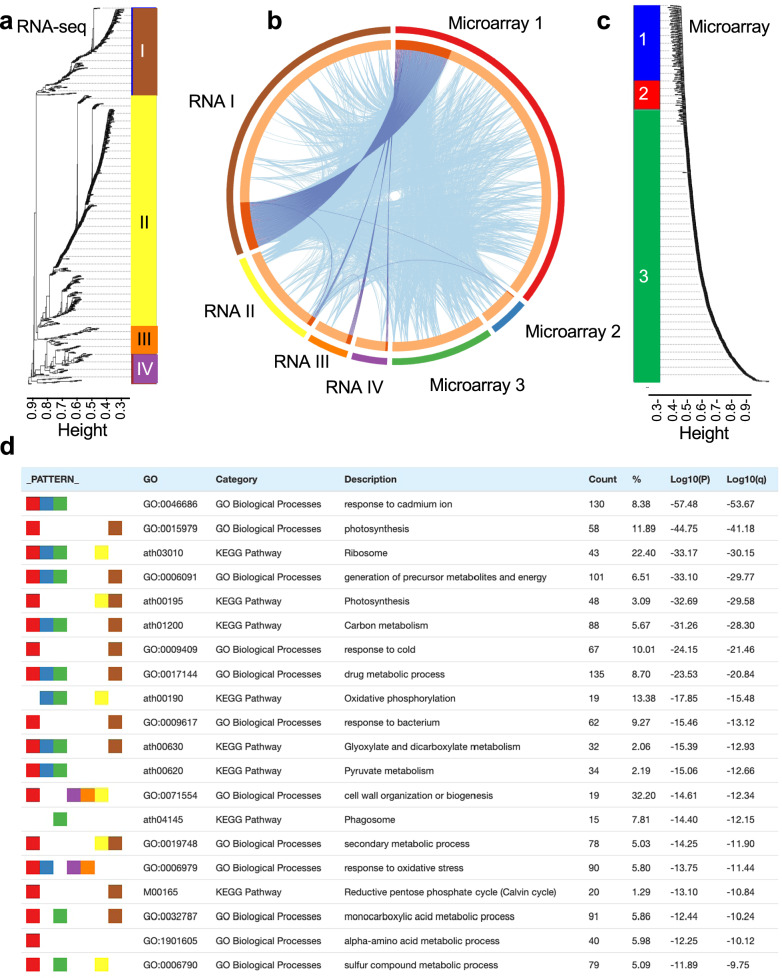
Unguided Weighted Gene Correlation Network Analysis (WGCNA) clustering of the Arabidopsis datasets used in this study. This analysis was performed on the DEGs identified in the RNA-seq (**a**) and microarray (**c**) datasets from the spaceflight experiments imported into the Matrix (see Table 2 for specific datasets used). This analysis identified 4 clusters of DEGs within the RNA-seq (**a**) and 3 clusters within the microarray analyses (**c**). **b** Overlap in the DEGs within each cluster between the WGCNA RNA-seq and microarray analyses. Purple curves link identical genes and light blue curves link genes that, although not identical, belong to the same enriched Gene Ontology term found in each clade. The inner circle represents gene lists, where hits are arranged along the arc. Genes that hit multiple clusters are colored in dark orange, and genes unique to a single cluster are shown in light orange. **d** List of top 20 significantly enriched Gene Ontologies drawn from the clusters of DEGs depicted in **a**–**c** that are shared by 2 or more clusters. The full list of enriched Gene Ontology terms is reproduced in Supplementary Data Fig. 1. Multiple colors under the PATTERN column indicate a pathway or process that is shared across multiple microarray or RNA-seq clades as denoted by their color coding in **a** and **c**. Count number of loci included in enrichment analysis, % proportion of all query genes that are found in the given Gene Ontology term, P *p*-value, q *p*-value adjusted for multiple testing. Analysis made using Metascape^24^.

Insights from the network of connections between the spaceflight-related datasets in the Matrix were then used to make comparisons between gene expression patterns seen in spaceflight treatments and ground control samples (e.g., excluding the ground-based spaceflight analog studies). Having defined the assay type (microarray versus RNA-seq) as one of the most important confounding factors when comparing spaceflight responsive transcripts across multiple datasets (Fig. 2), the microarray and RNA-seq datasets were separated into two parallel analysis pipelines. The data of the DEGs within the two series of datasets was then analyzed using Weighted Gene Co-expression Network Analysis (WGCNA). Unguided WGCNA clustering identified 3 groupings within the microarray datasets and 4 within the RNA-seq data (Fig. 5a–c). Krishnamurthy et al.^17^ have compared microarray and RNA-seq analyses of identical samples from Arabidopsis roots, concluding that although the two approaches broadly agreed (on ~66% of ~6400 DEGs in their study), RNA-seq analysis revealed significantly more DEGs. Thus, in our study the RNA-seq is likely providing a broader dataset within which to find enriched Gene Ontologies likely leading to the increased number of groupings found by our analysis.

The top 20 enriched ontology groupings shared between the RNA-seq and microarray analyses are summarized in Fig. 5d and the full set of significantly enriched Gene Ontologies is shown in Supplementary Fig. 2. Clade I in both the microarray and RNA-seq stands out as sharing the most common significantly DEGs. When expanding this analysis to include shared significantly enriched Gene Ontology terms (Fig. 5c), terms that broadly cover response to environmental stresses (such as to light, cold and bacteria) are seen (Supplementary Fig. 2). This observation supports the conclusions from numerous previous spaceflight analyses that plants exhibit a suite of stress-related responses when encountering the spaceflight environment. However, a further prominent and novel shared element seen across these analyses is changes in the expression of genes related to photosynthesis, other aspects of primary metabolism and also changes to secondary metabolism (Fig. 5d). This observation suggests that spaceflight and spaceflight-related treatments are likely impacting these fundamental aspects of plant function. However, as a note of caution, this analysis combines the responses across all the diverse plant datasets within the Matrix. Even though the analysis in Fig. 5d is filtered to exclude the broadest Gene Ontology terms (i.e., those terms encompassing more than 100 genes), such wide-ranging analysis might be expected to reveal only the most general common responses, whilst being relatively insensitive to more subtle or specific spaceflight responses. This is because of the variation likely imposed by the wide range of experimental designs encompassed by these datasets, i.e., in addition to spaceflight-related effects a host of other responses are likely superimposed on the data, diluting the signal from some spaceflight responses. It seems likely a similar reason explains the observation that, although there are shared spaceflight enriched gene ontologies between experiments, there is no individual DEG common to all these experiments. The Matrix facilitates a more targeted subset of comparisons between datasets (e.g., chosen based on commonalities in the hardware or plant samples used within each experiment) that might be expected to reduce this experimental design-driven noise to reveal these more specific shared gene groupings. An example of such an analysis described in the following section.

### Analysis of studies using common hardware: BRIC datasets provide 2 tissue types and 2 transcriptome assay types for meta-analysis

The analyses in Figs. 2 and 3 suggest that both the specific flight hardware used and its associated lighting regime significantly impact the patterns of gene expression noted in plants in spaceflight. Further, our network analyses (Fig. 3b) show that GLDS-17, GLDS-37, GLDS-38, GLDS-44, and GLDS-121 are all highly connected, especially for these factors. Thus, these studies all used etiolated seedlings grown in the dark in the Petri Dish Fixation Unit (PDFU) cassettes of the BRIC hardware. Additionally, samples were harvested at the young seedling stage of development (up to 12 days old) and all included a paired on-orbit and ground control design to allow for exploration of spaceflight-related patterns of DEGs^18–22^. Differences between the studies include ecotype and analysis type (microarray versus RNA-seq). Nevertheless, their high levels of similarity, especially at the level of the hardware and lighting used, suggested to us that they could provide an important set of similarly designed experimentation to help more robustly reveal common spaceflight responses. Additionally, all these studies have published in-house analyses from their respective research groups, allowing us to further test the relative merits of comparisons drawn between the in-house results from each original publication versus the common analytical pipeline that we have used in this study. Table 1 presents a summary of the total numbers of DEGs detected in these analyses at *p* ≤ 0.01 alongside the overlap in these gene lists between the in-house and common pipeline analyses (the full lists of DEGs are shown in Supplementary Data 4).

**Table 1:** Comparison of the differentially expressed gene counts from in-house and common pipeline analyses.

GeneLab Accession	Assay	GeneLab count	Original count	Loci in both	Difference	In-house reference
GLDS-17	Microarray	2459	499	34	1960	^37^
GLDS-44	Microarray	4031	3826	2597	205	^41^
GLDS-121	Microarray	2122	2177	2121	–55	^40^
GLDS-37	RNA-seq	2785	2084	927	701	^38^
GLDS-38	RNA-seq	3870	2919	2404	951	^39^

Data is taken from the original spaceflight research publications (in-house, i.e., using the original authors’ analyses with *p* ≤ 0.01) and the GeneLab analysis (*p* ≤ 0.01, adjusted for multiple hypothesis testing using the Benjamini and Hochberg method).

**Fig. 6:**
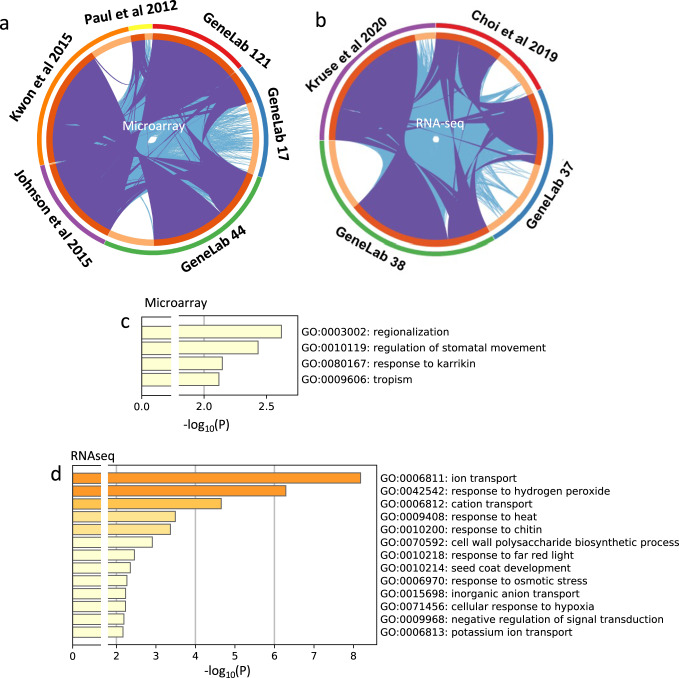
Analysis of shared DEGs between the in-house and GeneLab pipeline analyses of plant experiments performed in spaceflight using the BRIC hardware. Overlap between gene lists for microarray studies (**a**) or RNA-seq (**b**) where purple curves link identical genes and light blue includes the shared Gene Ontology term level. Curves link genes that belong to the same enriched Gene Ontology term. The inner circle represents gene lists, where hits are arranged along the arc. Genes that hit multiple lists are colored in dark orange, and genes unique to a list are shown in light orange. Sectors denoted by GeneLab ## show the analysis using GeneLab common pipeline; sectors denoted by a citation show the original authors’ in-house analysis. **c**, **d** Significantly enriched GO terms from analysis of common genes found in the microarray (**c**) and RNA-seq (**d**) analyses identified in both the in-house and GeneLab pipelines. Analysis in **c** and **d** performed using Metascape.

The five datasets were separated into microarray (three studies) and RNA-seq (two studies) groups and analyzed using the GeneLab pipelines outlined above before making an overall comparison across all the BRIC experiments. The significance threshold to identify DEGs was set at *p* < 0.01. In a previous comparative analysis, Johnson et al.^23^ reported no common genes amongst the BRIC-16 mission microarray studies (GLDS-17, -44 and -121) when using a cutoff of *p* < 0.01 but also applying a threshold of 5-fold or greater for induction or repression in transcript level as measured on their microarray (to define the most strongly regulated genes). We therefore reanalyzed these microarray results using a pipeline similar to the original authors’ analyses (Affymetrix Express pipeline and the Probe Logarithmic Intensity Error (PLIER) approach for normalization^21^, with a significance setting *p* < 0.01) but now using no fold-change filtering (Supplementary Data 4) to be more analogous to the GeneLab analytical pipeline we have also applied. Using the in-house analysis by the researchers (GLDS-17^18^, GLDS-44^22^) and the reanalysis of GLDS-121^21^ using PLIER, the results of our comparisons across all the microarray studies conducted with wild-type seedlings identified 86 spaceflight-related DEGs found across all studies (Supplementary Data 5). The GeneLab reanalysis identified 114 loci in common between the 3 studies, including 85% of the genes from the in-house analysis. Analysis of the 75 DEGs identified in all 3 studies by both analytical techniques using MetaScape^24^ (Fig. 6) revealed enrichment in Gene Ontology terms including: regionalization, response to Karrikin (a plant stress response pathway triggered by volatiles originally found in smoke), regulation of stomatal movement and tropism. These latter two terms are particularly interesting as disruption of gravitropic growth is one of the predicted responses of plants growing in the microgravity environment of spaceflight. Although patterns of development seen in plants growing in space often show more randomized directional growth than on the ground, molecular evidence for altered tropic response reflected in the patterns of transcriptional changes observed in spaceflight has been less clear. Thus, the highlighting of tropisms in Fig. 6c suggests the use of the Matrix approach for analyzing the available data may help reveal these molecular signatures. Further, this analysis revealed that stomatal behavior may be affected by spaceflight. Factors such as reduced buoyancy-driven convection in microgravity would be predicted to alter gas exchange at the stomatal pore^5,8^, likely playing out as altered stomatal function. Again, this effect has been difficult to reliably detect in the transcriptional fingerprints of spaceflight responses, but the targeted analyses driven by insights from the Matrix appear able to reveal evidence for these previously cryptic patterns of molecular changes. Repeating this analysis made using Metascape^24^ but with DAVID (the Database for Annotation, Visualization and Integrated Discovery^25^) as an alternative, widely used tool to assess gene ontology enrichments agreed with the analysis outlined above and did not reveal any new significantly enriched ontology terms at *p* < 0.01. This observation suggests the Metascape analysis outlined in Fig. 6 is likely capturing most of the patterns of ontology enrichment in the data.

GLDS-37 and GLDS-38 represent the BRIC samples studied with RNA-seq, necessitating independent analysis from the microarray datasets discussed above. Again, we used data from the published in-house bioinformatics approaches^19,20^ and the GeneLab common analytical pipeline. Comparison of these approaches in the WT Col-0 samples in both datasets shows that at a threshold of *p* < 0.01, the common pipeline identified 701 and 951 new loci as showing altered expression in spaceflight from GLDS-37 and GLDS-38 respectively, or about 25% more loci than found in the original authors’ analysis (Table 1 and Supplementary Data 4). Comparing the GeneLab pipeline-based analysis with that of the original peer-reviewed publications indicates agreement on 927 (GLDS-37) and 2404 (GLDS-38) DEGs. Further, within this analysis, 164 loci were significantly differentially expressed in both GLDS-37 and GLDS-38 (Supplementary Data 6). Gene Ontology enrichment analysis of these spaceflight-responsive DEGs across both studies and in both the in-house and GeneLab analytical pipelines (Fig. 6d) revealed enrichment in responses such as to oxidative stress, heat shock and changes in cell wall dynamics that have been highlighted in multiple previous plant spaceflight transcriptome studies (e.g., refs. ^14,19,21,22,26,27^). Reanalysis with the Matrix approach was also able to reveal a fingerprint of hypoxia which has been predicted as an important factor impacting biology operating with the reduced convective gas movements inherent in a microgravity environment^6,7^ but which has previously proven difficult to observe in analyses of transcriptional responses of individual flight experiments using the BRIC. In addition, Gene Ontologies related to various aspects of ion transport are prominent in our analysis targeting future investigations focused on both anion and cation transport as likely to be a fruitful targets for further understanding the effects of spaceflight on plants.

Lastly, since processes associated with responses to spaceflight are still largely unknown, Supplementary Data 7 provides a list of spaceflight-responsive DEGs from this analysis that currently have no GO or KEGG annotation. These genes provide potential targets for study for novel processes triggered by plant growth in space.

**Fig. 7:**
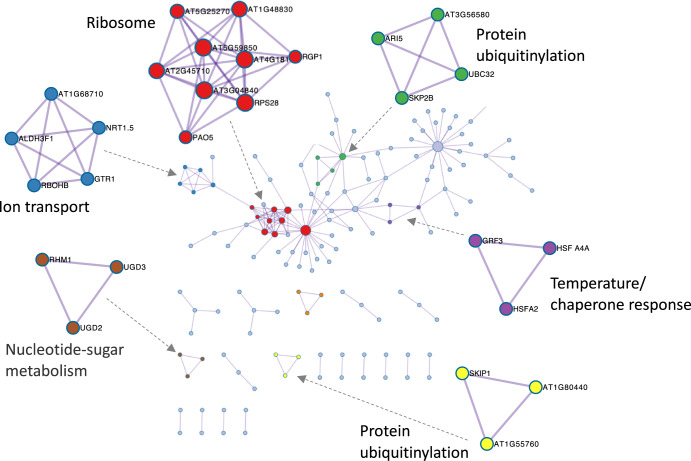
Protein:protein interaction network inferred from the common DEGs identified using the GeneLab analysis pipeline of GLDS-37 and GLDS-38. Analysis using Metascape with annotation of densely connected network elements identified with the MCode algorithm. Colors represent clusters grouped by shared ontology term. Size of circle shows the number of protein:protein interactions that each node/locus is annotated as being involved with as identified by the MCode analysis.

We next used Metascape analysis on these lists of DEGs from the analysis of GLDS-37 and GLDS-38 to explore potential protein:protein networks, applying Metascape’s protein-protein interaction enrichment analysis and Molecular Complex Detection (MCode)^28^. These analyses take the lists of differentially expressed genes and mine an array of protein interaction databases (STRING^29^, BioGrid^30^, OmniPath^31^, InWeb_IM^32^) for enriched networks of physical interactions. MCode then allows a focus on highly connected hubs when the numbers of proteins in the network become very high. These analyses again revealed enrichment for a response network associated with ion transport and chaperone activity (Fig. 7). In addition, multiple network clusters related to protein ubiquitinylation were identified. This observation suggests spaceflight may have triggered alterations in proteasome activity, possibly related to stress-induced protein turnover. Such a response to stress-related protein dysfunction would be consistent with the elevated chaperone activities suggested by the heat shock protein (HSP)-related protein:protein interaction cluster identified in this same analysis.

### Intersection between RNA and microarray analyses

**Fig. 8:**
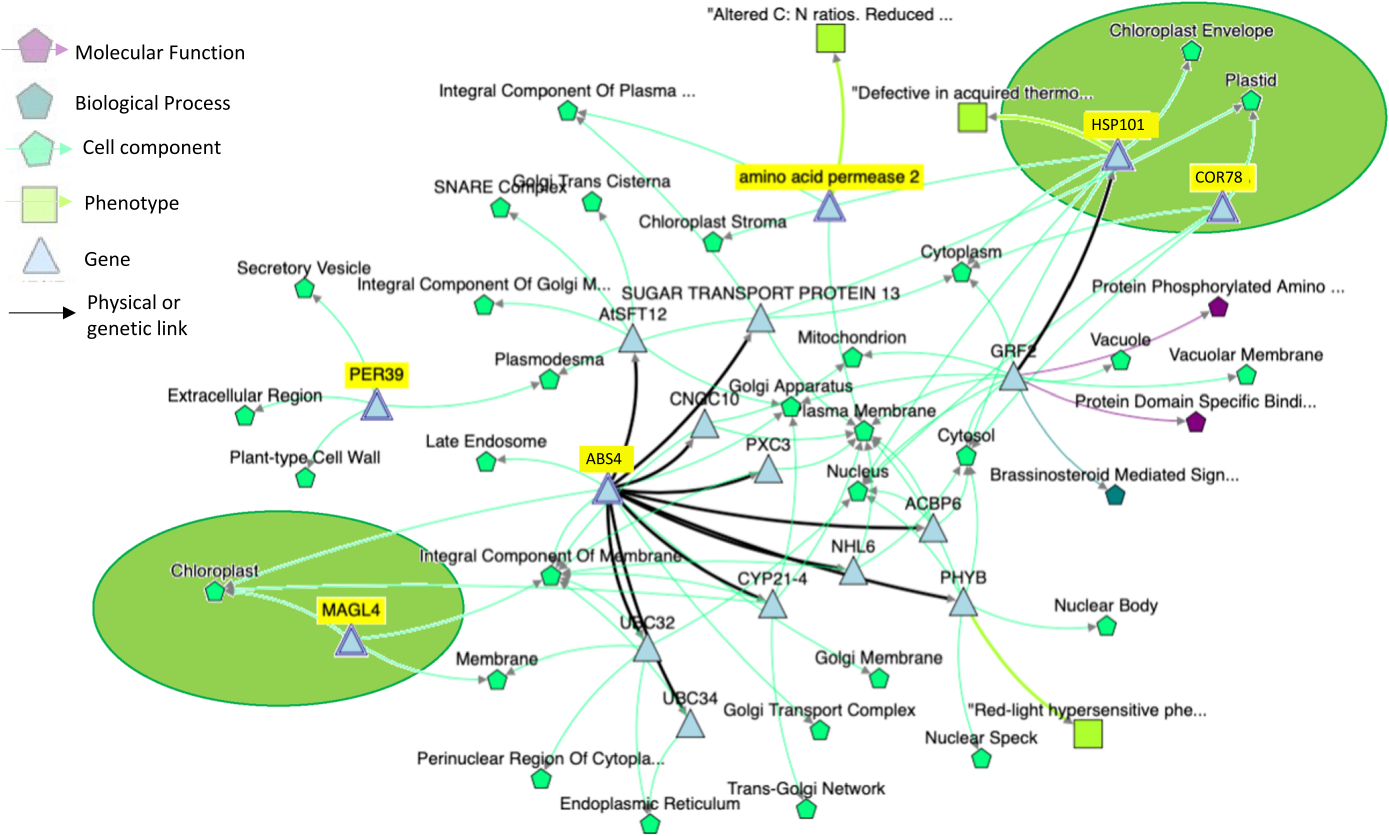
Network analysis of the 6 common spaceflight responsive genes identified from analysis of Arabidopsis seedlings flown in the BRIC hardware. Query genes are highlighted in yellow. AT1G74310 (*HOT1/HSP101*; *HEAT SHOCK PROTEIN 101*), AT1G58340 (*ABS4*, a plant MATE multidrug and toxic compound extrusion transporter), AT5G52310 (*COR78*; *COLD REGULATED 78*), AT4G11290 (*PRX39*, *PEROXIDASE 39*, a cell wall peroxidase), AT5G09220 (*AAP2, AMINO ACID PERMEASE 2*), and AT1G73480 (*MAGL4*, an α-β hydrolase family protein). Analysis performed using KnetMiner. Purple connector, link to biochemical function; cyan connector, link to physical location in cell; green connector, link to associated phenotype; black connector, direct physical or genetic linkage. Note links to plastid (green oval) for *MAGl4*, *HSP101* and *COR78*. An interactive version of this analysis is available at: https://knetminer.com/beta/knetspace/network/970c571c-15da-4b93-87ad-ef1418ef9d29.

By combining the differentially expressed gene lists from both microarray and RNA-seq analyses identified using the GeneLab common pipeline approach, 6 common spaceflight response loci were identified but in only three of the studies (GLDS-37, GLDS-38, and GLDS-121). This observation reinforces the idea that variation in experimental design and analysis approach may be obscuring some common patterns of response (see below). Within the BRIC-19 experiment that generated the data in GLDS-37, in addition to the Col-0 ecotype (also used in GLDS-38) and Ler-0 (also used in GLDS-121), two additional ecotypes were investigated (Cvi-0, Ws-2). These same six genes were also differentially expressed across all the ecotypes in this study, reinforcing their likely common response nature. These 6 common genes were: AT1G74310 (*HOT1/HSP101*; *HEAT SHOCK PROTEIN 101*), AT1G58340 (*ABS4*, a plant MATE multidrug and toxic compound extrusion transporter), AT5G52310 (*COR78*; *COLD REGULATED 78*), AT4G11290 (*PRX39*, a cell wall peroxidase), AT5G09220 (*AAP2, AMINO ACID PERMEASE 2*), and AT1G73480 (*MAGL4*, an α-β hydrolase). Analysis of these loci using the graph-based network analysis tool KnetMiner^23^ revealed broad connections to the plastid and membrane function (Fig. 8).

Some of these genes have been discussed in the individual analyses originally published on each BRIC experiment(s)^18–22^. However, the power of the current meta-analysis lies in highlighting these particular genes as possible core markers of the spaceflight response across multiple experiments within the BRIC hardware and revealing a difference in GLDS-17 and GLDS-44. Interrogating the experimental design reveals that one obvious difference between GLDS-17 and the other BRIC investigations is that 3% (w/v) sucrose was used in the seedling media in GLDS-17 compared to 0.3–1% (w/v) in the other studies. Sucrose is generally added to the media of Arabidopsis seedlings to support the heterotrophic growth of the plants in the dark conditions in the BRIC. However, the higher sucrose in BRIC-17 [https://genelab-data.ndc.nasa.gov/genelab/accession/GLDS-17] was specifically added to facilitate comparisons between the seedlings in this experiment and a parallel set of cell cultures that required much higher sucrose for growth. The differences in gene expression between BRIC-17 [https://genelab-data.ndc.nasa.gov/genelab/accession/GLDS-17] seedlings and the other BRIC experiments then implies that changes in primary metabolism experienced by the etiolated seedlings in the BRIC may be an important factor in determining spaceflight related transcriptional responses, echoing the altered primary metabolism inferred from our meta-analysis across all the spaceflight datasets analyzed as part of the Matrix in Fig. 5. Such observations are especially relevant in the context of possible spaceflight-related hypoxia discussed above. Indeed, Loreti et al.,^33^ used microarray analysis in ground-based research to investigate the changes that take place in seedlings that experience low oxygen stress with or without the addition of external sucrose. Their analyses revealed that exogenous sucrose significantly alters patterns of anoxia-related transcriptional change. Thus, the increased sucrose concentration in the media found in GLDS-17 should dramatically affect the plant hypoxia response and so is likely to alter responses to this particular effect of the spaceflight environment.

Precisely why GLDS-44 also does not show the conserved transcriptional responses seen in GLDS-37, GLDS-38, and GLDS-121 is less obvious as its experimental design is very similar to these other BRIC-based experiments and it was flown side-by-side on the same mission as GLDS-121. However, subtle features such as the seed planting density differed between these studies and so effects of plant density and competition might be superimposed on these results. This analysis then highlights how understanding the feature(s) in these experiments responsible for the differences in expression pattern offers enormous potential to define factors with wide-ranging effects on the plant spaceflight response; i.e., the difference(s) between GLDS-44 and GLDS-121 and the other BRIC experiments clearly had dramatic effects on the patterns of spaceflight-related gene expression and so exploring how these studies differ in design should help define some key spaceflight-response related factors.

Looking at the shared DEGs between GLDS-37, GLDS-38, and GLDS-121 identifies *HSP101* as a common spaceflight response marker. Indeed, upregulation of Heat Shock Proteins (HSPs) in the spaceflight environment is well known^18,19,27,34,35^. Heat Shock Proteins are molecular chaperones associated with protecting and refolding proteins in response to cellular damage^36^. Consistent with the enriched clades corresponding to photosynthesis identified in Fig. 5, patterns of *HSP101* upregulation and its relationship to the chloroplast (Fig. 8), suggest that this protein may play an important role in ameliorating chloroplastic proteotoxic stress possibly resulting from spaceflight-induced production of reactive oxygen species (ROS) in the plastid. Indeed, the HSP100 family are known to be induced by abiotic stressors such as oxidative stress^37^ and have even been linked to tolerance to the proteotoxic damage caused by hypoxia^38^. Previous work^14,19^ has demonstrated a significant correspondence between patterns of gene expression altered by oxidative damage from the high light stress response on Earth (which is strongly linked to damaging levels of plastid ROS production) and the spaceflight-associated DEGs identified in the seedlings from BRIC experiments. Similarly, the large number of plastid genes responding to spaceflight identified in seedling samples from BRIC-16/GLDS-44^22^ reinforces the idea that this organelle may be an important site of spaceflight-induced responses. However, it is important to note that the BRIC experiments we have analyzed were all conducted under dark growth conditions. Therefore, the light-driven reactions of photosynthetic electron transport that are a major source of plastid ROS production on Earth are not responsible for these spaceflight-related effects and so the source of any spaceflight-triggered ROS production within the plastid remains to be defined.

Our meta-analysis using the common GeneLab analysis pipelines also highlights *COR78* as likely a part of a conserved transcriptional response of Arabidopsis on orbit in the BRIC hardware. Although originally identified as a cold induced transcript, *COR78* is now known to be highly inducible in response to a range of abiotic factors ranging from wounding and salt exposure to osmotic stress, drought and even the hypobaric (low pressure) environments predicted for future large scale, space-based plant growth facilities^39–41^. A common feature of all these stressors is that they trigger signaling through ROS and induce oxidative stress. Indeed, *COR78* expression is regulated through the same ROS-responsive transcriptional cascades (i.e., H_2_O_2_ responsive modulation through the DREB2A transcription factor) that modulates heat shock response elements such as *HsfA3*^42^, providing a possible link to the heat shock factor component of the spaceflight response. In a further tantalizing link between the chloroplast and *COR78* response, *COR78* expression is co-regulated with elements of the plastid antioxidant system and indeed, its expression is thought to be tightly linked to the levels of H_2_O_2_ processing by the plant^43^.

In summary, the GeneLab database is accumulating an ever-increasing number of datasets that investigate the transcriptional effects of spaceflight on early plant development. This aggregation of information, along with careful curation represents a powerful resource to begin to understand spaceflight responses in these organisms. Spaceflight imposes some commonly encountered and some unique challenges when comparing datasets. Thus, as with all large omics-level analyses, differences in protocols and analysis pipelines can impact the robustness of comparisons. However, spaceflight also leads to further challenges related to an often-restricted capacity for biological replication and with limitations on experimental design dictated by available spaceflight hardware. We have begun to address some of these issues by applying a common analytical pipeline for datasets and then constructing a matrix of metadata to allow for sorting and comparison across studies driven by their known similarities and differences. In this work we focused on two elements to highlight the potential of this approach: (1) making broad comparisons across the entire sets of data to draw conclusions about confounding variables that likely superimpose differences on spaceflight datasets, and (2) making analyses focusing on the commonly used BRIC hardware to help researchers understand the possibilities offered by designing comparative analyses in the context of the Matrix metadata. However, the possible comparisons guided by this Matrix are vast and so there remain many more opportunities for the research community to draw new insights from Matrix-focused analyses.

From the Matrix-driven exploration presented here, we found that: (1) comparisons across different transcriptome monitoring technologies (RNA-seq versus microarray) should be performed with great care as differences in the technology used can impose greater variation on results than the biological treatment (spaceflight versus ground control); (2) environmental conditions and hardware-related constraints in the experimental design produce smaller but also important differences that can confound interpretation of the spaceflight versus ground control comparisons; (3) when these factors are controlled for, comparisons across the breadth of spaceflight-related Arabidopsis experiments reveal alterations in general responses to environmental stresses, photosynthesis, and other elements of primary and secondary metabolism (Fig. 5). These broad areas provide targets for the generation of future models of how spaceflight may affect plant physiology and development. Our analysis of the BRIC hardware shows how with a more targeted approach, common response genes can be identified that then point to potentially core spaceflight responses. For example, the BRIC analysis strongly points to the plastid as a likely shared response site across many spaceflight experiments. The observations of conserved roles for *HSP101* and *COR78* suggest that a fundamental disruption of the ROS and/or antioxidant systems related to the plastid may be accompanying plant growth in space.

It is important to note limitations inherent in the approach we have applied to meta-analysis of these spaceflight datasets. Due to flight and hardware constraints, the spaceflight experiments collected for this meta-study were limited to young seedlings of Arabidopsis. Although some experiments have tested the viability of plants to reach mature and reproductive developmental stages these have generally not involved omics research. We must await the data from more studies throughout the phases of the plant life cycle to understand how well our developing insights from seedlings and young plants will apply to individuals at maturity. Similarly, we must await further studies on a wider array of plant species to extend these approaches beyond the plant most commonly grown in spaceflight, Arabidopsis.

One further limitation on our approach is that at present we manually curate the import of each experiment’s metadata into the Matrix. However, the GeneLab data repository has standardized its metadata formats for both current and future datasets offering us the opportunity to automate both import and curation. This automated approach will be facilitated through GeneLab’s automatic programming interface (API) which offers a program-accessible link to the metadata files. Continual updates to the Matrix will allow the power of inferences drawn to grow as quickly as the new plant spaceflight datasets are deposited.

Our analysis of the BRIC datasets suggests that focusing on a few hardware options that can then be the subject of multiple flight studies would greatly add to the power of such comparative omics-level analyses. Nevertheless, the results presented here offer the promise that as these experimental data become available, meta-analyses across the broad plant biology omics data landscape will provide a powerful approach to supplement the insights drawn from analyses focused on each individual study in isolation. The Matrix analysis presented herein provides a toolset to help expedite the development of such new investigations. Additionally, while the scope of potential hypotheses generated by these analyses is extensive, the current Matrix meta-analysis highlights three specific focus areas for future research that may prove particularly fruitful. These include: (1) studies examining the effect of variable light regimes on space grown plant productivity and physiology, (2) analyses aimed at determining the potential causes of altered redox activities in the plastids of space flown plants, and (3) experiments examining the function of *HSP101*, *ABS4*, *COR78*, *PRX39*, *AAP2*, and *MAGL4* in response to spaceflight stressors.

## Methods

### Assay pipelines and datasets

The GeneLab data repository currently holds the largest number of publicly accessible datasets of omics- (transcriptomics-, proteomics-, epigenomics- and genomics-) based studies of biological, spaceflight-related studies. For our analyses of plant responses using this resource we focused on the results assessing changes in the transcriptome as the most numerous kind of dataset available. We included such studies based on the minimal criteria that they: (1) were performed on the most widely used plant model species, *Arabidopsis thaliana* (which represents nearly all of the plant data currently deposited in GeneLab) and (2) had at least 3 biological replicates per treatment (to provide statistical rigor on subsequent analyses). A summary of the 15 studies (encompassing 10 microarray and 6 RNA-seq GeneLab Data Sets, or GLDS) that fulfill these requirements is presented in Table 2. These experiments were performed on missions run by NASA, the European Space Agency and the Chinese Space Agency. To ensure the greatest degree of comparability between results, all of the primary data was reanalyzed through common computational approaches developed by GeneLab and implemented in the Galaxy computing environment^44^. Briefly, the microarray analysis pipeline used the R/Bioconductor software package limma^45^ to perform differential gene expression analysis. Background correction by the Robust Multichip Average (RMA) method and between array normalization by the quantile method^46^ were performed through the Bioconductor Oligo package^47^. Gene level estimation was generated using the Maximum Interquartile Range method and annotations were added using the Annotation-Db class gene annotations specific to *Arabidopsis thaliana* from the Bioconductor repository (www.bioconductor.org). In cases where multiple probes mapped to the same gene ID, representative probes were selected with the highest mean normalized intensity across all samples. Differential gene expression analysis used the linear model fit from the limma R package to perform pairwise comparisons for all groups. For each probe set, the variance of mean signal intensities was estimated, improved by an empirical Bayes method for combining variances of probes showing similar variability, and the significance of the difference between the means was evaluated with a t-test to obtain *p*-values. *p*-values were also adjusted to *q*-values to account for possible errors introduced through multiple hypothesis testing using the Benjamini and Hochberg method^48^ and so control for the false discovery rate. Details of the code used to process each dataset are available at https://github.com/nasa/GeneLab_Data_Processing/tree/master/Microarray/1-channel_arrays/GLDS_Processing_Scripts. Both the raw and processed data can be downloaded at https://genelab-data.ndc.nasa.gov/genelab/projects.

**Table 2:** Studies used in developing the plant transcriptional Matrix.

Accession	Study title	Assay type	Refs.
GLDS-7	The Arabidopsis spaceflight transcriptome: a comparison of whole plants to discrete root hypocotyl and shoot responses to the orbital environment	Microarray	^44^
GLDS-17	Transcription profiling by array of the response of Arabidopsis cultivar Columbia etiolated seedlings and undifferentiated tissue culture cells to the spaceflight environment	Microarray	^37^
GLDS-37	Comparison of the spaceflight transcriptome of four commonly used *Arabidopsis thaliana* ecotypes (Col, Ws, Ler and Cvi)	RNA-seq	^38^
GLDS-38	Proteomics and transcriptomics analysis of Arabidopsis seedlings in microgravity	RNA-seq	^39^
GLDS-44	Transcriptomics analysis of etiolated *Arabidopsis thaliana* seedlings in response to microgravity	Microarray	^41^
GLDS-46	Gamma radiation and HZE treatment of seedlings in Arabidopsis	Microarray	^60^
GLDS-120	Genetic dissection of the spaceflight transcriptome responses in plants: are some responses unnecessary?	RNA-seq	^50^
GLDS-121	Biological Research in Canisters-16 (BRIC-16): investigations of the plant cytoskeleton in microgravity with gene profiling and cytochemistry	Microarray	^40^
GLDS-136	Dissecting low atmospheric pressure stress: transcriptome responses to the components of hypobaria in Arabidopsis	Microarray	^61^
GLDS-147	Arg1 functions in the physiological adaptation of undifferentiated plant cells to spaceflight	Microarray	^51^
GLDS-205	HSFA2 functions in the physiological adaptation of undifferentiated plant cells to spaceflight microgravity environment	Microarray	^62^
GLDS-208	Comparative gene expression analysis in the *Arabidopsis thaliana* root apex using RNA-seq and microarray transcriptome profiles	Microarray and RNA-seq	^36^
GLDS-213	A whole-genome microarray study of *Arabidopsis* cell cultures exposed to microgravity for 5 days on board of Shenzhou 8	Microarray	^63^
GLDS-218	Spaceflight-induced alternative splicing during seedling development in *Arabidopsis thaliana*	RNA-seq	^64^
GLDS-251	RNA-seq analysis of the response of *Arabidopsis thaliana* to fractional gravity under blue-light stimulation during spaceflight	RNA-seq	^65^

In the table, the reference column denotes the initial publication on the data with the authors’ in-house analyses, when available. Datasets are publicly available at the GeneLab data repository using the url: https://genelab-data.ndc.nasa.gov/genelab/accession/GLDS-#/, where # represents the GLDS accession number for each study.

The RNA-seq analysis pipeline used the universal RNA-seq aligner STAR v2.7.1a^49^ and the RNA-Seq by Expectation Maximization approach (RSEM v1.3.1)^13,50^ along with the TAIR10 genome assembly^51^ accessed through Ensembl Plants^52,53^. Raw sequence data were trimmed and filtered with Trim Galore! (v0.6.2). The *Arabidopsis thaliana* Ensembl reference genome TAIR10, release 44, and respective GTF file were used to align trimmed reads with STAR (v2.7.1a) then the aligned reads were quantified using RSEM (v1.3.1). Quantification data was imported to R (v3.6.0) using the tximport package (v1.14.0) and normalized using the DESeq2 (v1.26.0) median of ratios method^54^. Differential expression analysis was performed with DESeq2 (v1.26.0) and pairwise comparisons of all groups were performed using the Wald test to generate *p*- and adjusted *p*-values, and the likelihood ratio test was used to generate the F statistic *p*-value. Gene annotations were assigned using the Bioconductor org.At.tair.db (v3.8.2), STRINGdb (v1.24.0)^29^, and PANTHER.db (v1.0.4)^55^ packages. Processing code for each RNA-seq dataset are available at https://github.com/nasa/GeneLab_Data_Processing/tree/master/RNA-seq/GLDS_Processing_Scripts and both the raw and processed data are deposited at https://genelab-data.ndc.nasa.gov/genelab/projects.

The associated metadata for each dataset was aggregated using a combination of the information provided alongside each GeneLab data submission, parallel manual curation from the literature and through interviews with the primary researchers. The Matrix of this data is available as both Supplementary Data 1 and as an interactive exploration environment developed in the Qlik database management software environment (Qlik Technologies Inc., King of Prussia, PA, USA) at https://gilroy-qlik.botany.wisc.edu/a/sense/app/20aa802b-6915-4b1a-87bd-c029a1812e2b.

When they have been employed in the data analyses, online tools such as the TOAST X-Species Transcriptional Explorer (https://gilroy-qlik.botany.wisc.edu/a/sense/app/ab2250b5-ee3a-4da8-b5da-fe87d5f2dbe6/overview), KnetMiner^23^, Metascape^24^, Ensembl GO^53^, the Kyoto Encyclopedia of Gene and Genomes^56^, AraCyc^57^ and Reactome^58^ are noted in the text and figure legends. Principal Component Analysis (PCA), Multidimensional Scaling analysis (MDS), t-distributed Stochastic Neighbor Embedding (T-SNE), Weighted Gene Correlation Network Analysis (WGCNA) and K-means statistical analyses were performed using the iDEP.94 R-package^59^. For these analyses, the normalized counts were imported from the GeneLab data repository and processed using R-studio. The R programming language provides for the statistical analysis of data (https://www.r-project.org/about.html) within a commercial development environment called R-studio (R-Studio inc. Boston, MA, USA).

### Reporting summary

Further information on research design is available in the Nature Research Reporting Summary linked to this article.

## Supplementary information

Supplemental Material

Supplementary Data 1

Supplementary Data 2

Supplementary Data 3

Supplementary Data 4

Supplementary Data 5

Supplementary Data 6

Supplementary Data 7

Reporting Summary

## Data Availability

Source data for this study are publicly available in the GeneLab data repository (https://genelab-data.ndc.nasa.gov/genelab/projects/) under the Accession codes GLDS-7; GLDS-17; GLDS-37; GLDS-38; GLDS-44; GLDS-46; GLDS-120; GLDS-121; GLDS-136; GLDS-147; GLDS-205; GLDS-208; GLDS-213; GLDS-218; GLDS-251.

## Code availability

Details of the code used to process each dataset are available at https://github.com/nasa/GeneLab_Data_Processing/tree/master/Microarray/1-channel_arrays/GLDS_Processing_Scripts. Both the raw and processed data can be downloaded at https://genelab-data.ndc.nasa.gov/genelab/projects. R scripts used for raw data processing, iDEP.92 analysis and visualization are available at https://github.com/dr-richard-barker/The-Matrix-2022 The Matrix of this data is available as both Supplementary Data 1 and as an interactive exploration environment developed in the Qlik database management software environment (Qlik Technologies Inc., King of Prussia, PA, USA) at https://gilroy-qlik.botany.wisc.edu/a/sense/app/20aa802b-6915-4b1a-87bd-c029a1812e2b.
