# Supplementary material for: Meta-analysis of the space flight and microgravity response of the Arabidopsis plant transcriptome
Source: NPJ Microgravity. 2023 Mar 20;9:21. doi: 10.1038/s41526-023-00247-6 (PMC10027818; doi:10.1038/s41526-023-00247-6)
Supplement: Supplementary file 1 — Supplemental Material [file 41526_2023_247_MOESM1_ESM.pdf]

Supplementary Materials for

The Matrix: Meta-analysis of the space flight and microgravity response of the Arabidopsis plant transcriptome

Richard Barker, Colin P.S. Kruse, Christina Johnson, Amanda Saravia-Butler, Homer Fogle, Hyun-seok Chang, Ralph Møller Trane, Noah Kinscherf, Alicia Villacampa, Aránzazu Manzano, Raúl Herranz, Laurence B. Davin, Norman G. Lewis, Imara Perera, Chris Wolverton, Parul Gupta, Pankaj Jaiswal, Sigrid S. Reinsch, Sarah Wyatt and Simon Gilroy.

Simon Gilroy

E-mail: sgilroy@wisc.edu

This PDF file includes:

Fig. S1
Fig. S2

Other supplementary materials for this manuscript include the following:

Supplementary Data 1-7

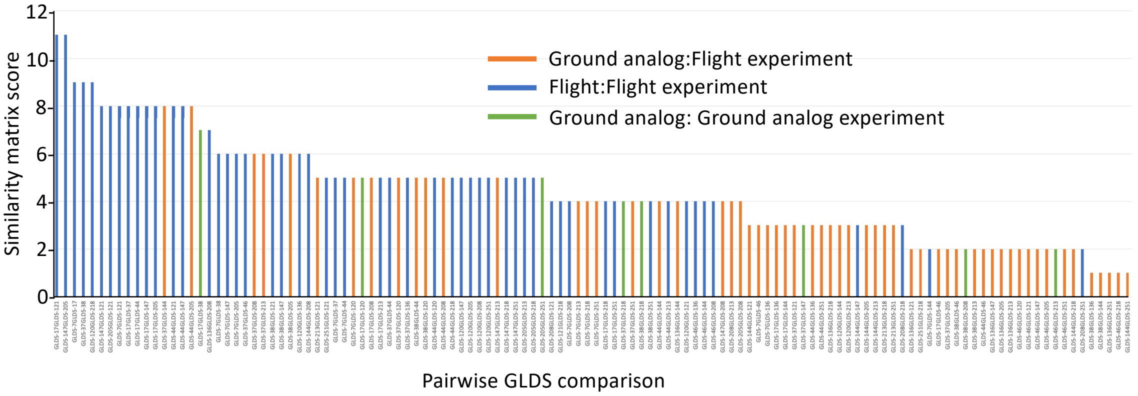

Supplementary Figure 1. Pairwise similarity scores of spaceflight-to-spaceflight, spaceflight-to-ground analog and ground analog -to-ground analog studies. Similarity scores were drawn from the similarity matrix in Supplementary Data Table 2. Orange, Flight mission study vs ground analog study; Blue, flight mission vs flight mission; Green, ground analog study vs ground analog study. Note, in this analysis, spaceflight studies are most similar to other spaceflight studies and least similar to ground analog experiments, which in turn are most similar to each other.

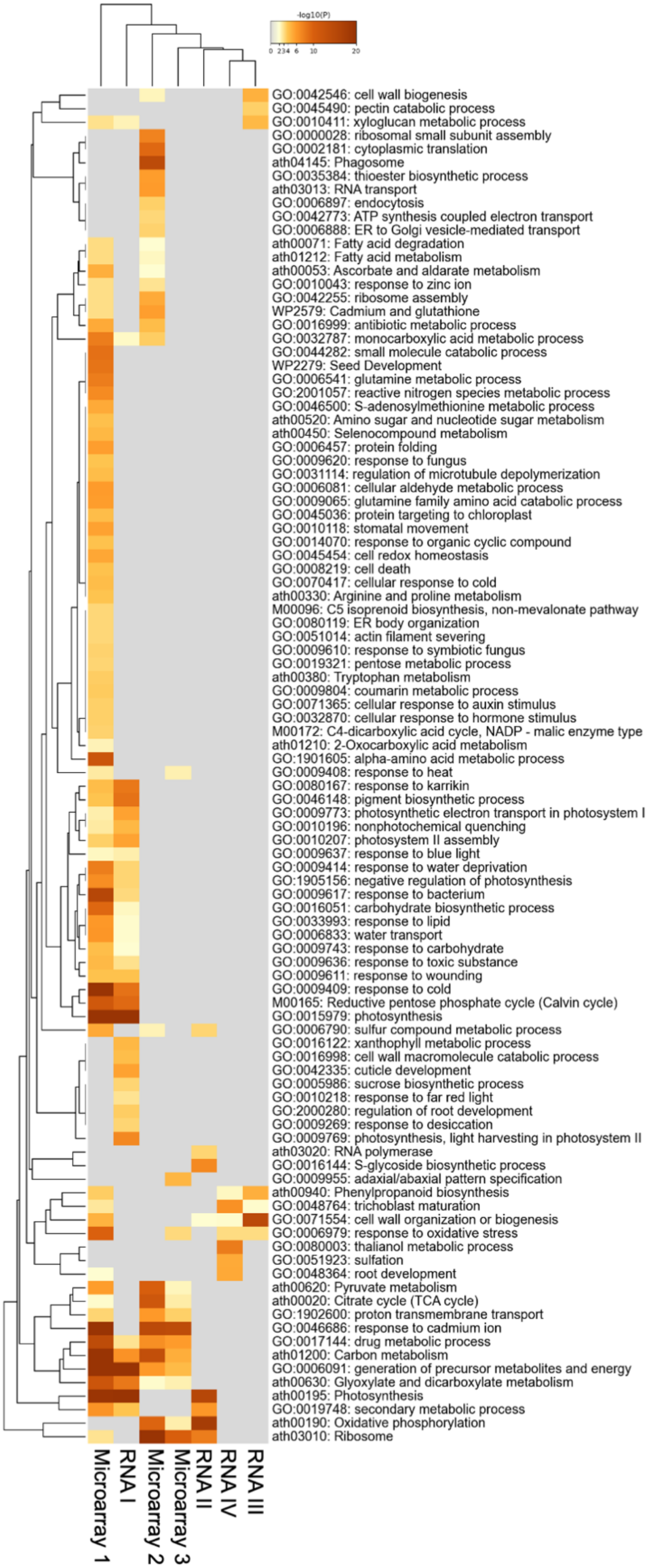
-log10(P)
0 234 6 10 20
GO:0042546: cell wall biogenesis
GO:0045490: pectin catabolic process
GO:0010411: xyloglucan metabolic process
GO:0000028: ribosomal small subunit assembly
GO:0002181: cytoplasmic translation
ath04145: Phagosome
GO:0035384: thioester biosynthetic process
ath03013: RNA transport
GO:0006897: endocytosis
GO:0042773: ATP synthesis coupled electron transport
GO:0006888: ER to Golgi vesicle-mediated transport
ath00071: Fatty acid degradation
ath01212: Fatty acid metabolism
ath00053: Ascorbate and aldarate metabolism
GO:0010043: response to zinc ion
GO:0042255: ribosome assembly
WP2579: Cadmium and glutathione
GO:0016999: antibiotic metabolic process
GO:0032787: monocarboxylic acid metabolic process
GO:0044282: small molecule catabolic process
WP2279: Seed Development
GO:0006541: glutamine metabolic process
GO:2001057: reactive nitrogen species metabolic process
GO:0046500: S-adenosylmethionine metabolic process
ath00520: Amino sugar and nucleotide sugar metabolism
ath00450: Selenocompound metabolism
GO:0006457: protein folding
GO:0009620: response to fungus
GO:0031114: regulation of microtubule depolymerization
GO:0006081: cellular aldehyde metabolic process
GO:0009065: glutamine family amino acid catabolic process
GO:0045036: protein targeting to chloroplast
GO:0010118: stomatal movement
GO:0014070: response to organic cyclic compound
GO:0045454: cell redox homeostasis
GO:0008219: cell death
GO:0070417: cellular response to cold
ath00330: Arginine and proline metabolism
M00096: C5 isoprenoid biosynthesis, non-mevalonate pathway
GO:0080119: ER body organization
GO:0051014: actin filament severing
GO:0009610: response to symbiotic fungus
GO:0019321: pentose metabolic process
ath00380: Tryptophan metabolism
GO:0009804: coumarin metabolic process
GO:0071365: cellular response to auxin stimulus
GO:0032870: cellular response to hormone stimulus
M00172: C4-dicarboxylic acid cycle, NADP - malic enzyme type
ath01210: 2-Oxocarboxylic acid metabolism
GO:1901605: alpha-amino acid metabolic process
GO:0009408: response to heat
GO:0080167: response to karrikin
GO:0046148: pigment biosynthetic process
GO:0009773: photosynthetic electron transport in photosystem I
GO:0010196: nonphotochemical quenching
GO:0010207: photosystem II assembly
GO:0009637: response to blue light
GO:0009414: response to water deprivation
GO:1905156: negative regulation of photosynthesis
GO:0009617: response to bacterium
GO:0016051: carbohydrate biosynthetic process
GO:0033993: response to lipid
GO:0006833: water transport
GO:0009743: response to carbohydrate
GO:0009636: response to toxic substance
GO:0009611: response to wounding
GO:0009409: response to cold
M00165: Reductive pentose phosphate cycle (Calvin cycle)
GO:0015979: photosynthesis
GO:0006790: sulfur compound metabolic process
GO:0016122: xanthophyll metabolic process
GO:0016998: cell wall macromolecule catabolic process
GO:0042335: cuticle development
GO:0005986: sucrose biosynthetic process
GO:0010218: response to far red light
GO:2000280: regulation of root development
GO:0009269: response to desiccation
GO:0009769: photosynthesis, light harvesting in photosystem II
ath03020: RNA polymerase
GO:0016144: S-glycoside biosynthetic process
GO:0009955: adaxial/abaxial pattern specification
ath00940: Phenylpropanoid biosynthesis
GO:0048764: trichoblast maturation
GO:0071554: cell wall organization or biogenesis
GO:0006979: response to oxidative stress
GO:0080003: thalianol metabolic process
GO:0051923: sulfation
GO:0048364: root development
ath00620: Pyruvate metabolism
ath00020: Citrate cycle (TCA cycle)
GO:1902600: proton transmembrane transport
GO:0046686: response to cadmium ion
GO:0017144: drug metabolic process
ath01200: Carbon metabolism
GO:0006091: generation of precursor metabolites and energy
ath00630: Glyoxylate and dicarboxylate metabolism
ath00195: Photosynthesis
GO:0019748: secondary metabolic process
ath00190: Oxidative phosphorylation
ath03010: Ribosome
Microarray 1
RNA I
Microarray 2
Microarray 3
RNA II
RNA IV
RNA III

Supplementary Figure 2. Unguided WGCNA clustering of the datasets within the Matrix defines 3 clades in the microarray data and 4 in the RNA-seq-based analyses. Significantly enriched ontology terms in each clade are shown. Analysis using Metascape. This data was used to generate the figure showing overlap in ontologies in Fig. 5b and the top 20 enriched ontologies are reproduced in Fig. 5d.
