# Supplementary material for: Meta-analysis of the space flight and microgravity response of the Arabidopsis plant transcriptome
Source: NPJ Microgravity. 2023 Mar 20;9:21. doi: 10.1038/s41526-023-00247-6 (PMC10027818; doi:10.1038/s41526-023-00247-6)
Supplement: Supplementary file 9 — Reporting Summary [file 41526_2023_247_MOESM9_ESM.pdf]

nature portfolio

Corresponding author(s): Simon Gilroy

Last updated by author(s): Nov 1, 2022

Reporting Summary

Nature Portfolio wishes to improve the reproducibility of the work that we publish. This form provides structure for consistency and transparency in reporting. For further information on Nature Portfolio policies, see our Editorial Policies and the Editorial Policy Checklist.

Statistics

For all statistical analyses, confirm that the following items are present in the figure legend, table legend, main text, or Methods section.

n/a	Confirmed	
☐	☒	The exact sample size (*n*) for each experimental group/condition, given as a discrete number and unit of measurement
☒	☐	A statement on whether measurements were taken from distinct samples or whether the same sample was measured repeatedly
☐	☒	The statistical test(s) used AND whether they are one- or two-sided<br>*Only common tests should be described solely by name; describe more complex techniques in the Methods section.*
☐	☒	A description of all covariates tested
☐	☒	A description of any assumptions or corrections, such as tests of normality and adjustment for multiple comparisons
☐	☒	A full description of the statistical parameters including central tendency (e.g. means) or other basic estimates (e.g. regression coefficient) AND variation (e.g. standard deviation) or associated estimates of uncertainty (e.g. confidence intervals)
☒	☐	For null hypothesis testing, the test statistic (e.g. *F*, *t*, *r*) with confidence intervals, effect sizes, degrees of freedom and *P* value noted<br>*Give P values as exact values whenever suitable.*
☒	☐	For Bayesian analysis, information on the choice of priors and Markov chain Monte Carlo settings
☒	☐	For hierarchical and complex designs, identification of the appropriate level for tests and full reporting of outcomes
☒	☐	Estimates of effect sizes (e.g. Cohen's *d*, Pearson's *r*), indicating how they were calculated

Our web collection on statistics for biologists contains articles on many of the points above.

Software and code

Policy information about availability of computer code

Data collection	Details of the code used to process each dataset are available at https://github.com/nasa/GeneLab_Data_Processing/tree/master/Microarray/1-channel_arrays/GLDS_Processing_Scripts. Both the raw and processed data can be downloaded at https://genelab-data.ndc.nasa.gov/genelab/projects.<br>R scripts used for raw data processing, iDEP.92 analysis and visualization are available at https://github.com/dr-richard-barker/The-Matrix-2022<br>The Matrix of this data is available as both Supplementary Data 1 and as an interactive exploration environment developed in the Qlik database management software environment (Qlik Technologies Inc., King of Prussia, PA, USA) at https://gilroy-qlik.botany.wisc.edu/a/sense/app/20aa802b-6915-4b1a-87bd-c029a1812e2b.
Data analysis	Commercial: QlikSense 2019 database management software (Qlik Technologies Inc., King of Prussia, PA, USA)

For manuscripts utilizing custom algorithms or software that are central to the research but not yet described in published literature, software must be made available to editors and reviewers. We strongly encourage code deposition in a community repository (e.g. GitHub). See the Nature Portfolio guidelines for submitting code & software for further information.

Data

Policy information about availability of data

All manuscripts must include a data availability statement. This statement should provide the following information, where applicable:

- Accession codes, unique identifiers, or web links for publicly available datasets
- A description of any restrictions on data availability
- For clinical datasets or third party data, please ensure that the statement adheres to our policy

The datasets analyzed for this study are publicly available in the GeneLab data repository (https://genelab-data.ndc.nasa.gov/genelab/projects/) under the Accession codes https://genelab-data.ndc.nasa.gov/genelab/accession/GLDS-###, where ### represents the Gene Lab Dataset number defined in Table 1.

Human research participants

Policy information about studies involving human research participants and Sex and Gender in Research.

Reporting on sex and gender	NA
Population characteristics	NA
Recruitment	NA
Ethics oversight	NA

Note that full information on the approval of the study protocol must also be provided in the manuscript.

Field-specific reporting

Please select the one below that is the best fit for your research. If you are not sure, read the appropriate sections before making your selection.

☒ Life sciences ☐ Behavioural & social sciences ☐ Ecological, evolutionary & environmental sciences

For a reference copy of the document with all sections, see nature.com/documents/nr-reporting-summary-flat.pdf

Life sciences study design

All studies must disclose on these points even when the disclosure is negative.

Sample size	This paper describes a tool for mining plant biology datasets at deposited at NASA's GeneLab Data repository. Experimental design was defined by the original authors' study but studies with insufficient replication (less than 3) were excluded.
Data exclusions	This paper describes a tool for mining Arabidopsis plant biology transcriptome data deposited at NASA's GeneLab Data repository. Plant datasets in the GeneLab repository were excluded only if they were not from Arabidopsis thaliana and if they lacked 3 or more biological replicates and so were not amenable to statistical analyses.
Replication	Datasets imported for analysis were limited to those with 3 or more biological replicates.
Randomization	This paper describes a tool for mining plant biology datasets at deposited at NASA's GeneLab Data repository. Experimental design was defined by the original authors' study.
Blinding	This paper describes a tool for mining plant biology datasets at deposited at NASA's GeneLab Data repository. Experimental design was defined by the original authors' study.

Reporting for specific materials, systems and methods

We require information from authors about some types of materials, experimental systems and methods used in many studies. Here, indicate whether each material, system or method listed is relevant to your study. If you are not sure if a list item applies to your research, read the appropriate section before selecting a response.

Materials & experimental systems

n/a	Involved in the study
☒	☐ Antibodies
☒	☐ Eukaryotic cell lines
☒	☐ Palaeontology and archaeology
☒	☐ Animals and other organisms
☒	☐ Clinical data
☒	☐ Dual use research of concern

Methods

n/a	Involved in the study
☒	☐ ChIP-seq
☒	☐ Flow cytometry
☒	☐ MRI-based neuroimaging
